# Supplementary material for: MetaRibo-Seq measures translation in microbiomes
Source: Nat Commun. 2020 Jun 29;11:3268. doi: 10.1038/s41467-020-17081-z (PMC7324362; doi:10.1038/s41467-020-17081-z)
Supplement: Supplementary file 10 — Supplementary Data 7 [file 41467_2020_17081_MOESM10_ESM.zip › File2/Confidence_High_Taxonomy/498_out.krona.html]

Javascript must be enabled to view this page.

members
magnitude
magnitudeUnassigned
count
unassigned
taxon
rank

498\_out

19

superkingdom
19
2

976
19
phylum

1
class
200643

171549
order
1

family
1
171552

838
1
genus

28135
1
species

SRS893232\_contig\_number\_31554

class
18
117743

200644
18
order

49546
18
family

18
genus
1016

712212
species
2

SRS015692\_contig\_number\_contig-100\_3054.109990SRS078431\_contig\_number\_35013


SRS024289\_contig\_number\_25927SRS058261\_contig\_number\_11391
327575
species
2


SRS011126\_contig\_number\_23035SRS012285\_contig\_number\_38722SRS014578\_contig\_number\_contig-100\_1580.149497SRS014690\_contig\_number\_4581SRS016200\_contig\_number\_2275SRS022149\_contig\_number\_12041SRS023538\_contig\_number\_11773SRS053895\_contig\_number\_8683SRS145509\_contig\_number\_3556
1019
9
species

1018
species
1

SRS105074\_contig\_number\_4068


SRS054430\_contig\_number\_11383SRS077022\_contig\_number\_2561SRS102814\_contig\_number\_14958SRS893298\_contig\_number\_9457
1017
4
species
